# Effects of Air Pollution on Hospital Emergency Room Visits for Respiratory Diseases: Urban-Suburban Differences in Eastern China

**DOI:** 10.3390/ijerph13030341

**Published:** 2016-03-19

**Authors:** Peng Liu, Xining Wang, Jiayin Fan, Wenxin Xiao, Yan Wang

**Affiliations:** 1 School of Environmental Science and Engineering, Shandong University, Jinan 250100, China; lp901012@gmail.com; 2 Shandong Center for Disease Control and Prevention, Jinan 250014, China; wangxn1@126.com; 3 Shandong Experimental High School, Jinan 250001, China; fanjiayin1999@sina.com; 4 School of Foreign Languages and Literature, Shandong University, Jinan 250100, China; legentracy@163.com

**Keywords:** air pollution, emergency room visits, respiratory diseases, urban-suburban discrepancies

## Abstract

A study on the relationships between ambient air pollutants (PM_2.5_, SO_2_ and NO_2_) and hospital emergency room visits (ERVs) for respiratory diseases from 2013 to 2014 was performed in both urban and suburban areas of Jinan, a heavily air-polluted city in Eastern China. This research was analyzed using generalized additive models (GAM) with Poisson regression, which controls for long-time trends, the “day of the week” effect and meteorological parameters. An increase of 10 μg/m^3^ in PM_2.5_, SO_2_ and NO_2_ corresponded to a 1.4% (95% confidence interval (CI): 0.7%, 2.1%), 1.2% (95% CI: 0.5%, 1.9%), and 2.5% (95%: 0.8%, 4.2%) growth in ERVs for the urban population, respectively, and a 1.5% (95%: 0.4%, 2.6%), 0.8% (95%: −0.7%, 2.3%), and 3.1% (95%: 0.5%, 5.7%) rise in ERVs for the suburban population, respectively. It was found that females were more susceptible than males to air pollution in the urban area when the analysis was stratified by gender, and the reverse result was seen in the suburban area. Our results suggest that the increase in ERVs for respiratory illnesses is linked to the levels of air pollutants in Jinan, and there may be some urban-suburban discrepancies in health outcomes from air pollutant exposure.

## 1. Introduction

The health effects of air pollution have been a matter of increasing public concern. Among all risk factors investigated in the 2010 Global Burden of Disease (GBD2010), outdoor air pollution ranked as the top global health risk burden and resulted in approximately 3.2 million premature annual deaths worldwide [1]. Abundant epidemiological studies have shown that severe air pollution episodes can have a bearing on daily mortality and hospital admissions for respiratory diseases and cardiovascular diseases [2,3]. It was estimated that the 1952 London smog episode caused approximately 12,000 excess deaths and 50%–300% higher mortality rates than the previous year [4]. During the years 1973 to 1980 in Philadelphia, U.S., a 100 μg/m^3^ increase in total suspended particulates (TSP) was associated with an increase of 7% (95% confidence internal (95% CI): 4%, 10%) in total mortality [5]. In Utah Valley, U.S., during the periods when daily PM_10_ concentrations exceeded 150 μg/m^3^, the increases in children's and adult hospital admissions for respiratory diseases were nearly 200 percent and 44 percent, respectively [6]. Comparable results were reported in research studies performed in Germany [7], Canada [8], Ireland [9] and Finland [10]. Some researchers also investigated the health effects of air pollution in developing countries. Gouveia and Fletcher found daily hospital admissions of children in São Paulo, Brazil, for total respiratory diseases had significant associations with O_3_, NO_2_ and PM_10_ [11]. Cropper *et al.* reported that a 100 μg/m^3^ increase in TSP result in a 2.3% increase in non-traumatic deaths in Delhi, India [12]. Loomis *et al.* estimated that a 10 µg/m^3^ in 24-h average PM_2.5_ level was related to a 4.2% (95% CI: 0.97%, 8.61%) increase in infant mortality in Mexico City [13]. Vichit-Vadakan *et al.* evaluated three panel studies in Bangkok, Thailand, and reported that an increase of 45 µg/m^3^ in PM_10_ was associated with about a 30% increase in lower respiratory symptoms in children and 50% increase in adults [14]. Wong *et al.* concluded that the city-combined effects of air pollutants in Asia were estimated to be equal or greater than those in developed countries [15].

However, low levels of ambient air pollutants are still significantly associated with health effects, e.g., chronic obstructive pulmonary diseases hospitalizations [16], and asthma hospital admissions [10]. Unlike hospital admissions, emergency room admissions are unrestricted by bed availability [17]. Currently, emergency rooms visits (ERVs) are recognized as a sensitive indicator for the short-term health outcomes of air pollution, including some severe cases of acute respiratory diseases and more severe cases.

In China, the unprecedented economic growth accompanied by industrialization and urbanization is based on enormous natural resource and energy consumption, of which coal still remains the largest share [18]. Moreover, a series of severe air pollution events in developed regions and megacities caused multiple effects upon industrial production activities, transportations and health as well as the living standards of citizens and rural residents. Recent studies performed in the metropolises, such as Beijing and Shanghai, have observed a statistically significant association between particulate matter, gaseous pollutants and mortality and hospital admissions for respiratory and cardiovascular diseases [19,20,21], attracting a great deal of attention from the government and the public. 

As a typical industrial city in Eastern China, Jinan was listed among the most heavily air-polluted cities in the world [22], and has been suffering from intense air pollution in the last two decades due to its high population density, large traffic volume and intense industrial activities [23,24]. It was reported that the annual average concentration of PM_2.5_ was 108 µg/m^3^ in Jinan in 2013, which is much greater than the safe level of 10 µg/m^3^ recommended by the World Health Organization (WHO). Previous researches have demonstrated that mortality was associated with increases in PM_2.5_ concentrations, even if the annual concentration was lower than 10 μg/m^3^ or if the daily concentration was lower than 30 μg/m^3^ [25,26]. However, there are rare studies on the potential health effects of air pollution in Jinan. Moreover, air pollution in suburban areas in China cannot be overlooked, and another feature of interest in this paper is to quantify the effects of outdoor air pollutants on the suburban residents.

Thus, in our study, the daily number of ERVs for respiratory diseases is used as a health outcome to analyze the short-term effects of PM_2.5_, SO_2_ and NO_2_ on the exposed population across urban and suburban areas, and to explore the urban-suburban differences.

## 2. Materials and Methods 

### 2.1. Study Area

Jinan (36°10’–37°90’ N, 116°12’–117°35’ E) is a semi-enclosed city surrounded by the Yellow River and Taishan Mountain, with an urban population of 3.75 million inhabitants. According to statistics, more than 21 million tons of coal was consumed in this city in 2012, emitting 114,520 tons of SO_2_ and 51,609 tons of industrial dust [27]. In addition, due to its unique landform and rapid growth in building construction sites and motor vehicles, Jinan has been suffering from severe air pollution and is listed as one of the major haze regions in China [23]. 

This study was performed in the Licheng District of Jinan, the largest district containing both urban and suburban areas. In the center of Licheng District, Hongjialou Subdistrict (HS) is a typically residential and commercial area characterized by convenient traffic, vast education resources, large apartment buildings, as well as a high population density of 18 thousand residents per square kilometer. Due to its unique representativeness, there have been several published papers in which the sampling sites were placed in HS to provide information about the exposure of the urban population to air pollution in Jinan [28,29,30]. According to the official land-use planning in Jinan, Zhonggong Town (ZT) is located in the suburb of Jinan [31]. Approximately 20 kM away from the urban areas, ZT, where many farmers live, is an area of tourism for outdoor activities and agricultural production with little urbanization and a low population density, and thus, it is able to reflect the air quality level in the suburban areas of Jinan. 

### 2.2. Data on Emergency Room Visits and Air Pollutants

Data on hospital ERVs (from January 2013 to December 2014) were collected from medical record databases in two public general hospitals in Licheng, one of which is the Traditional Chinese Medical (TCM) Hospital of Licheng in HS, and the other is the People’s Hospital of Licheng in ZT. The study samples acquired from the hospitals are geographically representative because two hospitals have regular patients from the corresponding areas. Next, according to the tenth revision of International Classification of Diseases Codes (ICD-10), the ERVs data for respiratory diseases were extracted, including pneumonia (ICD-10: J12–18), asthma (ICD-10: J45–J46), upper respiratory tract infection (ICD-10: J00–06), and chronic obstructive pulmonary disease (ICD-10:J40–44). 

Daily concentrations of PM_2.5_, SO_2_ and NO_2_ in HS were measured by one automated environmental monitoring station provided by the Environmental Protection Bureau of Jinan, and data on air pollutant concentrations in ZT were recorded by another station. Daily temperature (°C) and relative humidity during the study period were obtained from the China Meteorological Administration.

### 2.3. Statistical Analysis

**Figure A1:**
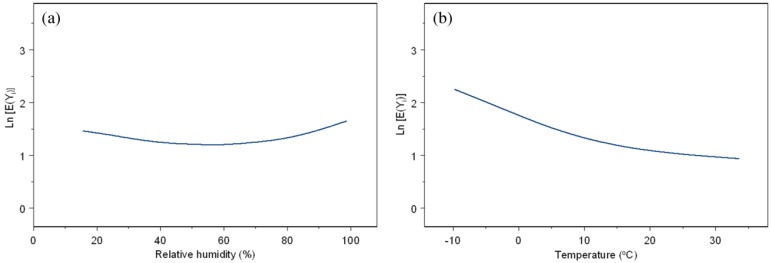
Smooth function curves of relative humidity and temperature: (**a**) relative humidity; (**b**) temperature.

Generalized additive models (GAM) with Poisson regression were constructed in R software with MGCV package to analyze the relationship between daily concentrations of air pollutants and the number of ERVs for respiratory diseases. Considering the confounding effects of long-term trends, seasonal patterns and meteorological parameters, the smoothing spline functions involved in calendar time, temperature and relative humidity were applied respectively. Using partial autocorrelation functions (PACF), the value of the degree of freedom (DF) for time trends was selected [32]. Based on Akaike’s information criterion (AIC), we specified the appropriate dfs in smoothing spline functions for weather conditions [19,33]. Further, the day of the week (DOW) was also introduced into the model. Before introducing the air pollutant factors, residuals of the models were examined in residual plots. Below was the final model:

(1) Log[E(Yi)]=intercept+βZi+DOW+s(time,df)+s(temperature,df)+s(humidity,df)

where E(Yi) represents the expected number of daily ERVs for respiratory diseases on day i; β indicates the regression coefficient; Zi is the daily concentration of air pollutant on day i; DOW is a categorical variable; s(time, df) denotes a smoothed function of calendar time with 7 df per year to control seasonality and longer-term trends; s(temperature, df) is a smoothed function of temperature with 3 df; and s(humidity, df) is a smoothed function of humidity with 3 df (Figure A1).

In view of the lag effects of air pollutants on health outcomes, a lag period ranging from 0 to 4 days prior to the occurrences of hospital emergency room visits (current day (lag0) up to 4 days before (lag4)) was adopted. The relative risk (RR) and its 95% confidence interval (CI) for a 10 μg/m^3^ increase of each pollutant were calculated. Statistical significance was considered only when the *p*-value was smaller than 0.05 in two-sided tests.

## 3. Results and Discussion

### 3.1. Descriptive Statistics

**Figure 1:**
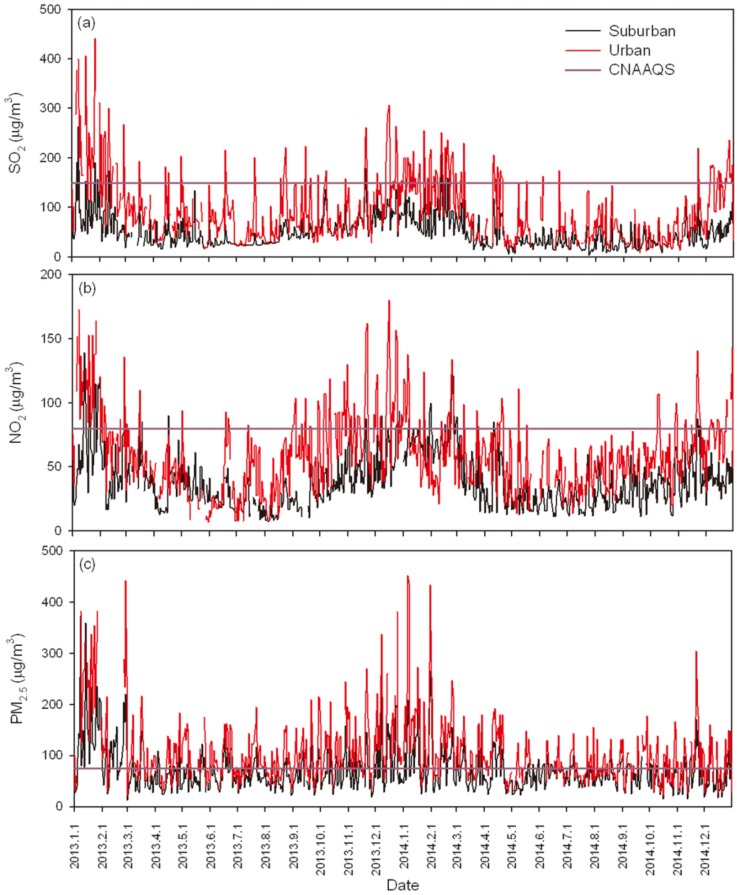
Time series distributions of SO_2_, NO_2_ and PM_2.5_ in Jinan during 2013 to 2014. The horizontal lines in (**a**); (**b**) and (**c**) represent the standard daily average limits of SO_2_ (150 μg/m^3^), NO_2_ (80 μg/m^3^) and PM_2.5_ (75 μg/m^3^) in Chinese National Ambient Air Quality Standards (CNAAQS), respectively.

The variations of the three air pollutants in the urban and suburban areas are shown in Figure 1. The high levels of PM_2.5_ concentration warrant attention. Comparatively speaking, the values of the air pollutants in both areas slightly decreased from 2013 to 2014, while the suburban area was less polluted than the urban area.

During the study period, there were 2625 ERVs for respiratory diseases registered in the hospital of HS and 2420 in ZT. The mean daily average concentrations of SO_2_, NO_2_ and PM_2.5_ in HS and ZT were 95.4 μg/m^3^, 60.0 μg/m^3^, 108.0 μg/m^3^ and 49.9 μg/m^3^, 38.9 μg/m^3^, 70.7 μg/m^3^, respectively (Table 1). According to Class II of the Chinese National Ambient Air Quality Standards (CNAAQS) used in residential urban and rural areas (annual average value: 60 μg/m^3^ for SO_2_, 40 μg/m^3^ for NO_2_ and 35 μg/m^3^ for PM_2.5_), it was found that all three of the pollutants in the urban area were 0.5–2 times higher than the standard limits, while only PM_2.5_ exceeded the standard limit in the suburb, indicating that the urban population represents a group more exposed to ambient air pollution than the suburban population. Based on CNAAQS (daily mean concentrations: SO_2_ = 150 μg/m^3^, NO_2_ = 80 μg/m^3^, PM_2.5_ = 75 μg/m^3^), the exceeding standard ratio (ESR) of each pollutant was calculated during 2013 and 2014, that is, the ratio of the number of days when the daily concentration of the pollutant is above the standard value to the total number of study days (Table 1). The ESR of SO_2_ (20.3%) was nearly the same as NO_2_ (20.4%) in the urban area, while the value of SO_2_ (1.6%) was lower than NO_2_ (5.7%) in the suburban area. Among the three air pollutants, PM_2.5_ pollution was more serious than NO_2_ and SO_2_ in both areas, and the ESR in the urban area (67.2%) was nearly twice as much as that in the suburbs (33.9%). Obviously, air pollution in the urban area of Jinan was more severe than that in the suburban area, particularly PM_2.5_.

The correlations between air pollutants and meteorological parameters are shown in Table 2. In the urban area, SO_2_, NO_2_ and PM_2.5_ were moderately correlated with each other (r = 0.563–0.677), and inversely correlated with average temperature (r = −0.534, −0.626 and −0.251, respectively). Relative humidity was positively correlated with NO_2_ and PM_2.5_ (r = 0.123 and 0.354, respectively), and inversely correlated with SO_2_ (r = −0.059). In the suburban area, the extent of correlations among air pollutants and weather conditions showed a similar pattern to the urban area; however, the correlation coefficients between SO_2_, NO_2_ and PM_2.5_ were found to be higher, which indicated that the emission sources of air pollutants in the suburb trended to be more similar and concentrated than those in the urban area of Jinan.

### 3.2. One-Pollutant Models

**Figure 2:**
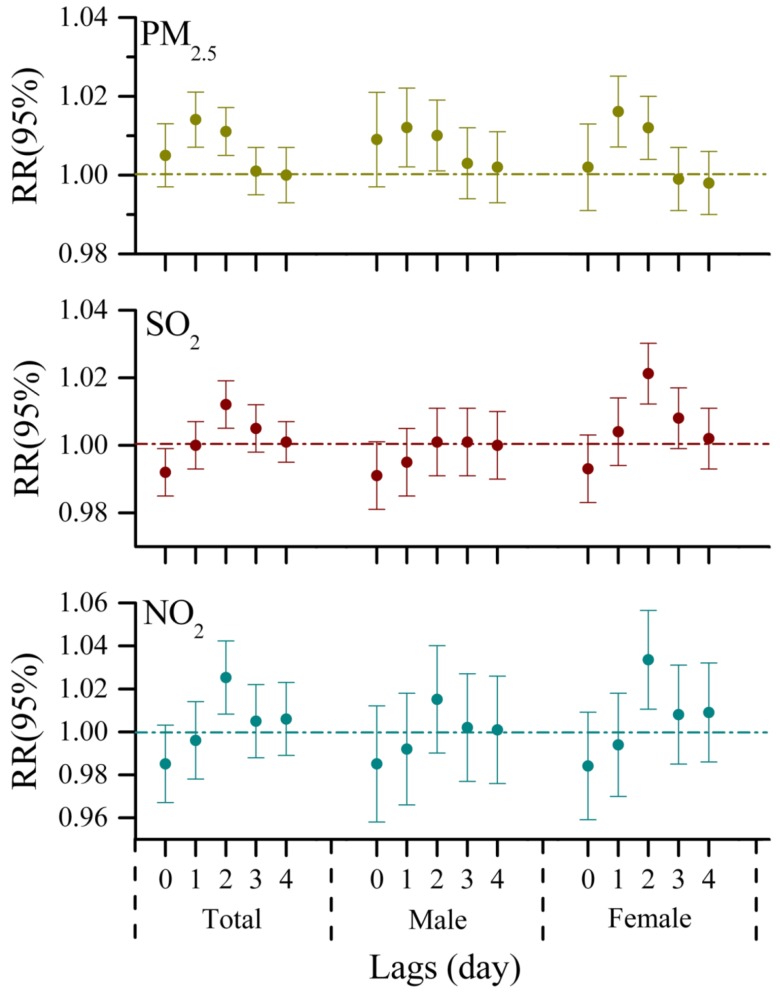
Relative risks (RRs with 95% CI) of hospital emergency room visits for respiratory complaints in association with per 10μg/m^3^ increase in PM_2.5_, SO_2_ and NO_2_ for the urban population of Jinan at different lags (lag0, lag1, lag2, lag3, lag4).

By adjusting for time trend, day of the week, temperature, and relative humidity in time-series analyses, the effects of three air pollutants on the daily number of ERVs at each lag day for the urban population were observed in Figure 2. For the total population, exposure to air pollutants on the current day did not contribute to the number of ERVs for respiratory diseases immediately, and the relative risks for ERVs were gradually weak with a lag of 3 and 4 days. PM_2.5_ was positively and significantly associated with ERVs at lag1 and lag2, and a 1.4% (95% CI: 0.7%, 2.1%) increased risk of ERVs due to respiratory complaints was associated with a 10 μg/m^3^ increment in PM_2.5_ one day before (Table 3). While the significant risk to ERVs for respiratory diseases was only strongly associated with 2-day lagged exposure to SO_2_ and NO_2_, an increase of 10 μg/m^3^ in SO_2_ and NO_2_ two days before predicted an increase of 1.2% (95% CI: 0.5%, 1.9%) and 2.5% (95% CI: 0.8%, 4.2%) in the number of ERVs for respiratory diseases, respectively. When the analysis was stratified by gender, the significant effects of PM_2.5_ on visits to hospital emergency rooms at lag1 were observed in both female and male groups (1.6%, 95% CI: 0.7%, 2.5%; 1.2%, 95% CI: 0.2%, 2.2%, respectively), whereas the strong influence of SO_2_ and NO_2_ existed in females but not in males, indicating that females in urban areas are more susceptible to air pollution than males, which is consistent with the results of other studies conducted in urban areas [19,34].

**Figure 3:**
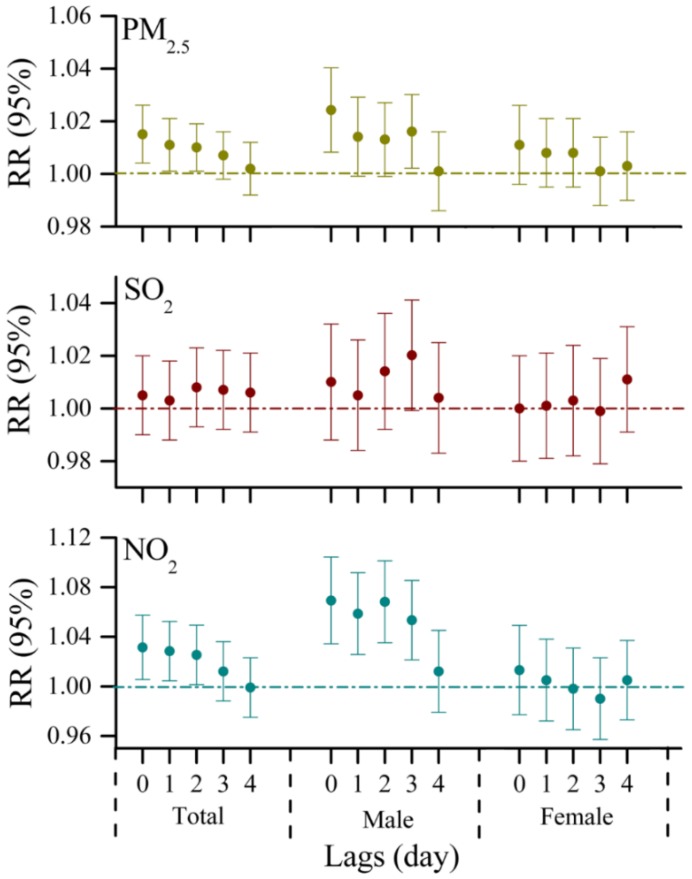
Relative risks (RRs with 95% CI) of hospital emergency room visits for respiratory complaints in association with per 10 μg/m^3^ increase in PM_2.5_, SO_2_ and NO_2_ for the suburban population of Jinan at different lags (lag0, lag1, lag2, lag3, lag4).

However, there are several differences in the lag effects of air pollutants in ZT. For the total population in ZT, PM_2.5_ and NO_2_ had similar lag patterns with a lag of 0, 1, and 2 days, and there was no evidence that SO_2_ was associated with visits to emergency rooms for respiratory diseases during the entire lag days examined (Figure 3). A 10 μg/m^3^ increase in PM_2.5_ and NO_2_ corresponded with an increase of 1.5% (95% CI: 0.4%, 2.6%) and 3.1% (95% CI: 0.5%, 5.7%) in ERVs for respiratory complaints, respectively (Table 3). Regarding the gender differences, we also stratified the analyses for associations between the variations of air pollutant levels and the daily number of ERVs. For males, remarkable increases in the risk of ERVs for respiratory diseases due to exposure to PM_2.5_ and NO_2_ in 0–3 days lag models, and the strongest positive associations were observed on the current day. An increase of 2.4% (95% CI: 0.8%, 4.0%) and 6.9% (95% CI: 3.4%, 10.4%) in the number of ERVs for respiratory complaints was associated with a 10 μg/m^3^ increase in PM_2.5_ and NO_2_, respectively. In contrast, no significant relationships between air pollutants and visits to emergency rooms for respiratory problems were found in females (Figure 3). Compared with the results obtained from the urban area, we observed some gender differences in the health effects of air pollution between the two populations. This finding could be attributed to different socioeconomic levels, as characterized by education, occupation and income, which are reported to be a factor for respiratory diseases [35,36]. In China, residents of suburban areas usually have lower socioeconomic levels, and spend more time in outdoor activities and less time in air conditioning than those in urban areas [37], and thus they are likely to have relatively high exposures to air pollution. In addition, cigarette smoking habit and body mass index (BMI) also have a prevalence of respiratory symptoms [38,39]. However, due to a lack of information, the reasons for this discrepancy are not completely clear and warrant further investigation.

In this study, it was found that estimated effects of PM_2.5_ on the ERVs for respiratory diseases in the urban and suburban populations were very similar in one-pollutant models. Regarding gaseous pollutants, NO_2_ appeared to have a stronger effect on the ERVs for respiratory diseases than SO_2_ in both areas. This finding was also observed by some epidemiological and biological studies [16,40,41]. Inhalation of sulfur dioxide (SO_2_) leads to rapid-onset bronchoconstriction, and a greater decrease in pulmonary function in asthmatic subjects than healthy subjects [42], potentially resulting from a TNF-α promoter polymorphism identified in asthmatic patients [43]. Personal exposure to nitrogen dioxide (NO_2_) has also been found to increase the severity of an asthma exacerbation [44], and induce inflammation in the airways as indicated by neutrophil influx and reduced lymphocyte subpopulations [45]. In addition, NO_2_ might increase the effects of an inhaled allergen as a sensitizing agent [46].

### 3.3. Muti-Pollutant Models

In one-pollutant models, strong associations with the daily number of ERVs were found in the total population of both areas. Considering the colinearity among air pollutants, we examined the relationships in multi-pollutant models with combinations of SO_2_, NO_2_ and PM_2.5_.

**Figure 4:**
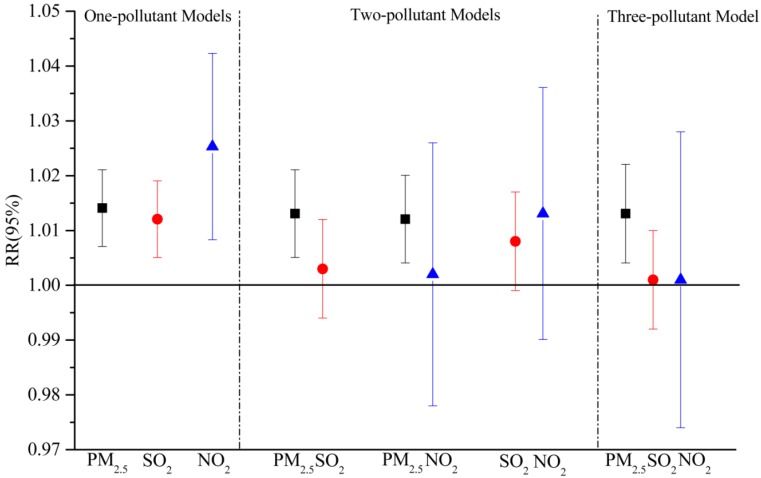
Adjusted estimated relative risks (RRs with 95% CI) of emergency room visits due to respiratory complaints with per 10 μg/m^3^ increase in PM_2.5_, SO_2_ and NO_2_ in multi-pollutant models for the urban population in Jinan.

The results of multi-pollutant models for the urban population are shown in Figure 4. After the adjustment for PM_2.5_, the associations between gaseous pollutants and daily ERVs were not statistically significant, whereas the estimated effects of PM_2.5_ observed for respiratory emergency room visits were not substantially attenuated when SO_2_ and (or) NO_2_ were included in the models. Similar to other studies on urban air pollution, our findings showed that PM_2.5_ remained a significant association with the morbidity of respiratory diseases after controlling for confounding factors, such as long-term trend and weather conditions as well as other gaseous pollutants. This finding indicated that PM_2.5_ could be a strong predictor of hospital emergency room visits for respiratory diseases in urban areas, and similar results were reported in other cities [47,48]. 

**Figure 5:**
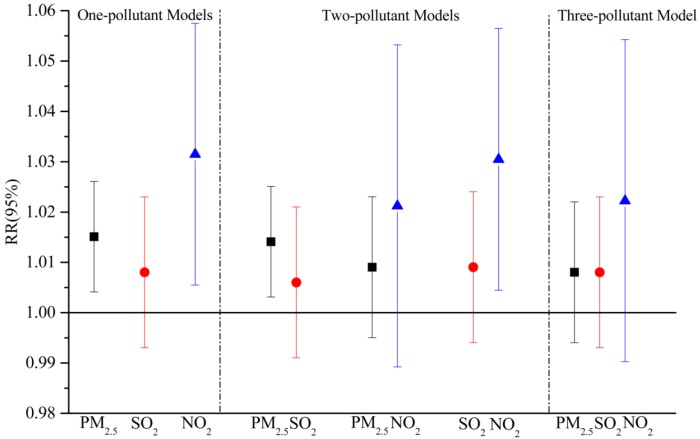
Adjusted estimated relative risk (RRs with 95% CI) of emergency room visits due to respiratory complaints with per 10 μg/m^3^ increase in PM_2.5_, SO_2_ and NO_2_ in muti-pollutant models for the suburban population in Jinan.

However, some different results were found in the suburban area (Figure 5). After adjusting for SO_2_ in the two-pollutant model, PM_2.5_ had a significant relationship with ERVs for respiratory diseases, while the estimated effect of PM_2.5_ was no longer statistically significant by the addition of NO_2_. Similarly, the relative risk of NO_2_ evaluated in the two-pollutant models with SO_2_ was 1.030 (95% CI: 1.004, 1.056), while a positive but insignificant association with NO_2_ were observed after an adjustment for PM_2.5_ [1.022, 95% CI (0.990, 1.054)]. In addition, in the three-pollutant model, there was no evidence observed that PM_2.5_, SO_2_ or NO_2_ had significant effects on the daily number of ERVs for respiratory diseases, which was also found in some other studies [49,50]. One potential explanation is the correlation coefficients between SO_2_, NO_2_ and PM_2.5_ in the suburban area were relatively higher than those in the urban area (Table 2), and those air pollutants could have more combined effects.

## 4. Conclusions

On the basis of a time series analysis of the relationship between air pollution and hospital emergency room visits (ERVs) for respiratory diseases in the urban and suburban areas of Jinan from 2013 to 2014, we observed that ERVs for respiratory illnesses were significantly associated with the levels of air pollutants and further corroborated that ERVs for respiratory diseases can be used as a sensitive indicator for health outcomes of air pollution. Females were found to be more susceptible to air pollution than males in the urban area when the analysis was stratified by gender, and the reverse trend was observed in the suburban area. Furthermore, air pollutants in the suburban area could have more combined effects on ERVs for respiratory illnesses than those in the urban area. Our findings indicate that there may be some urban-suburban discrepancies in the health outcomes of air pollutant exposure. Further studies need to be conducted in suburban areas to determine these discrepancies. 

## Figures and Tables

**Table 1:** Summary of daily air pollutants during 2013–2014 (μg/m^3^).

Area	Pollutant	Mean ± SD	Min	25%th	50%th	75%th	Max	ESR ^1^ (%)
Urban	SO_2_	95.4 ± 67.3	9.0	45.0	76.0	129.0	456.0	20.3
	NO_2_	60.0 ± 29.6	7.0	39.0	57.0	76.0	180.0	20.4
	PM_2.5_	108.0 ± 64.6	21.0	67.0	91.0	132.0	452.0	67.2
Suburban	SO_2_	49.9 ± 32.4	4.0	26.0	41.0	66.0	263.0	1.6
	NO_2_	38.9 ± 21.5	8.0	22.0	34.0	49.0	140.0	5.7
	PM_2.5_	70.7 ± 41.2	13.0	44.0	60.0	84.0	373.0	33.9

^1^ ESR: exceeding standard ratio.

**Table 2:** Pearson correlation coefficients between outdoor air pollutants and meteorology parameters in the urban and suburban areas of Jinan.

Pollutant	SO_2_	NO_2_	PM_2.5_	Temperature
NO_2_	0.677 * (0.706 *) ^1^			
PM_2.5_	0.563 * (0.656 *)	0.628 * (0.659 *)		
Temperature	−0.534 * (−0.534 *)	−0.626 * (−0.466 *)	−0.251 * (−0.289 *)	
Relative humidity	−0.059 (0.008)	0.123 * (−0.001)	0.354 * (0.259 *)	0.129 * (0.129 *)

^1^ The correlation coefficients in the suburb are in parentheses; * *p* < 0.05.

**Table 3:** Percent increase (95% CI) of hospital emergency room visits for respiratory diseases with a 10 μg/m^3^ increase in PM_2.5_, SO_2_ and NO_2_ in one-pollutant models for the urban and suburban populations in Jinan.

Area	Pollutant	Total (%)	Female (%)	Male (%)
Urban	PM_2.5_	1.4 (0.7, 2.1)	1.6 (0.7, 2.5)	1.2 (0.2, 2.2)
	SO_2_	1.2 (0.5,1.9)	2.1 (1.2, 3.0)	0.1 (−0.9, 1.1)
	NO_2_	2.5 (0.8, 4.2)	3.4 (1.1, 5.7)	1.5 (−1.0, 4.0)
Suburban	PM_2.5_	1.5 (0.4, 2.6)	1.1 (−0.4, 2.6)	2.4 (0.8, 4.0)
	SO_2_	0.8 (−0.7, 2.3)	1.1 (−0.9, 3.1)	2.0 (−0.1, 4.1)
	NO_2_	3.1 (0.5, 5.7)	1.3 (−2.3, 4.9)	6.9 (3.4, 10.4)

## Abbreviations

The following abbreviations are used in this manuscript:

PM_2.5_	Particulate matter less than 2.5 μm in diameter
ERVs	Emergency room visits
RR	Relative risk
HS	Hongjialou Subdistrict
ZT	Zhonggong Town
